# Effects of heliox and non-invasive neurally adjusted ventilatory assist (NIV-NAVA) in preterm infants

**DOI:** 10.1038/s41598-021-95444-2

**Published:** 2021-08-04

**Authors:** Natalia Neumann-Klimasińska, T. Allen Merritt, Jennifer Beck, Izabela Miechowicz, Marta Szymankiewicz-Bręborowicz, Tomasz Szczapa

**Affiliations:** 1 Neonatal Biophysical Monitoring and Cardiopulmonary Therapies Research Unit, Department of Neonatology, Poznan University of Medical Sciences, Poznan, ul. Polna 33, 60-535 Poznan, Poland; 2 Loma Linda University School of Medicine, Loma Linda, CA USA; 3 Department of Critical Care, Keenan Research Centre for Biomedical Science of St. Michael’s Hospital, St. Michael’s Hospital, Toronto, ON M5B1W8 Canada; 4 Department of Pediatrics, University of Toronto, Toronto, Canada; 5 Institute for Biomedical Engineering and Science Technology (iBEST), St-Michael’s Hospital, Ryerson University, Toronto, Canada; 6 Department of Computer Science and Statistics, Poznan University of Medical Sciences, Poznan, Poland

**Keywords:** Respiratory distress syndrome, Paediatric research

## Abstract

Due to its unique properties, helium–oxygen (heliox) mixtures may provide benefits during non-invasive ventilation, however, knowledge regarding the effects of such therapy in premature infants is limited. This is the first report of heliox non-invasive neurally adjusted ventilatory assist (NIV-NAVA) ventilation applied in neonates born ≤ 32 weeks gestational age. After baseline NIV-NAVA ventilation with a standard mixture of air and oxygen, heliox was introduced for 3 h, followed by 3 h of air-oxygen. Heart rate, peripheral capillary oxygen saturation, cerebral oxygenation, electrical activity of the diaphragm (Edi) and selected ventilatory parameters (e.g., respiratory rate, peak inspiratory pressure) were continuously monitored. We found that application of heliox NIV-NAVA in preterm infants was feasible and associated with a prompt and significant decrease of Edi suggesting reduced respiratory effort, while all other parameters were stable throughout the study, and had similar values during heliox and air-oxygen ventilation. This therapy may potentially enhance the efficacy of non-invasive respiratory support in preterm neonates and reduce the number of infants progressing to ventilatory failure.

## Introduction

Since the discovery of helium in nineteenth century, it has found numerous applications in medicine^1^. Its low density and diffusion coefficient make helium a particularly valuable asset in respiratory care^2^. According to Bernouilli's law, the low density of this gas directly affects the change in type and velocity of flow in the respiratory tract^2^. It may influence the respiratory function in premature infants as they are characterized by very small diameters of the airways and thus high respiratory resistance^3^. In the narrow airway, helium–oxygen mixture (heliox) can enhance the gas flow by reducing turbulence. In such setting, the airway pressure during ventilation with constant flow of heliox, will be lower in comparison to standard (air-oxygen) mixture ventilation. As the diffusion coefficient of carbon dioxide in the alveoli filled with helium–oxygen mixture is higher than with air, heliox ventilation can additionally improve the gas exchange^4^.

Conventional ventilation with heliox (helium and oxygen mixture) was found to reduce work of breathing, improve oxygenation and CO_2_ elimination, as well as enhance the distribution of gas within the respiratory system^2^. Findings from previous studies on heliox application in newborns during mechanical ventilation are encouraging^5–7^. However, knowledge about the physiological effects of this gas mixture during the non-invasive ventilation (NIV) in premature infants is limited.

Non-invasive neurally adjusted ventilatory assist (NIV-NAVA) provides a unique type of synchronization with patient’s respiratory effort. The electrical activity of the diaphragm (Edi)—recorded via miniaturized sensors on the baby’s nasogastric feeding tube—is used to control the timing and amplitude of assist during NAVA. The assist is achieved by adjusting the NAVA level, a conversion factor between the Edi and the delivered assist^8^. NIV-NAVA has been found safe and feasible, even in the most vulnerable and immature neonates^9^. Apart from ventilatory support NAVA enables a quantitative assessment of Edi, which can serve as a valuable diagnostic tool^10^. High Edi values reflect increased respiratory effort. Provision of the adequate respiratory support results in the decrease of Edi voltage^11^.

Synchronization of nasal intermittent positive pressure ventilation (NIPPV) may improve outcomes in premature infants with RDS and reduce extubation failure^12^. In premature infants synchronized NIPPV was associated with greater decrease in the work of breathing (WOB) than nCPAP alone^13^. NIV-NAVA proved more efficacious than both non-synchronized NIPPV and nCPAP after extubation^14,15^. The combination of heliox and NIV-NAVA may promote NIV success, providing a synergistic approach to premature infants with respiratory failure. Hence, the aim of the study was to assess the influence of heliox augmented NIV-NAVA in premature infants with respiratory failure on selected physiological and clinical parameters, evaluating gas exchange and diaphragmatic function.

## Results

23 premature infants born in 2017–2018 were included in the study. In 12 neonates NIV was utilized as the primary respiratory support and in 11 infants it was used post-extubation. The mean duration of pregnancy was 29 weeks GA and mean birth weight 1396 g. The rate of antenatal steroid therapy in the studied group was low as compared with the data from the region^16^. However, the studied cohort included also outborn infants transferred from non-tertiary centers, possibly with reduced use of antenatal steroids. Two patients from the group receiving NIV as primary support required intubation and surfactant administration after the study. Two neonates enrolled post-extubation required reintubation within 12 h after the study. Table 1 presents characteristics of the studied group.

**Table 1:** Patients’ characteristics.

Birth weight (g) mean (SD)	1396 (525)
Gestational age (weeks) mean (SD)	29 (3)
Day of life mean (SD)	8 (8)
Apgar score 1’ median (range)	6 (2–9)
Apgar score 5’ median (range)	8 (4–9)
Multiple birth n (%)	7 (30)
Prenatal steroids n (%)	14 (61)
Chorioamnionitis n (%)	4 (17)

**Figure 1:**
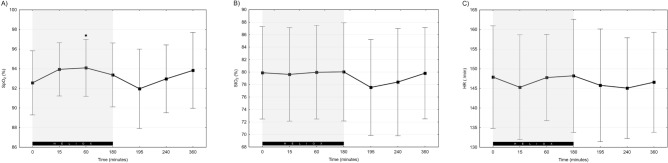
Values of SpO_2_ (**A**), B) StO_2_ (**B**) and HR (**C**) during the study. Grey background highlights heliox NIV-NAVA period. Data presented as mean ± SD; *indicates statistically significant changes versus baseline.

Infants’ general condition was stable throughout all phases of the study. The average SpO_2_ in both groups ranged from 92 to 94%. There was a subtle but statistically significant increase in SpO_2_ after 60 min of heliox ventilation (92.6 vs 94.1%, *p* = 0.032). Termination of heliox administration was associated with a small (93.4 vs 92.0%; *p* = 0.389), transient decrease in SpO_2_ (Fig. 1A). Cerebral oxygenation during the study ranged from mean StO_2_ of 70 to 88% (Fig. 1B). The statistical analysis of StO_2_ and HR measured in selected timepoints did not show any significant differences between study phases (Fig. 1B,C) (*p* = 0.156, *p* = 0.778, respectively).

**Figure 2:**
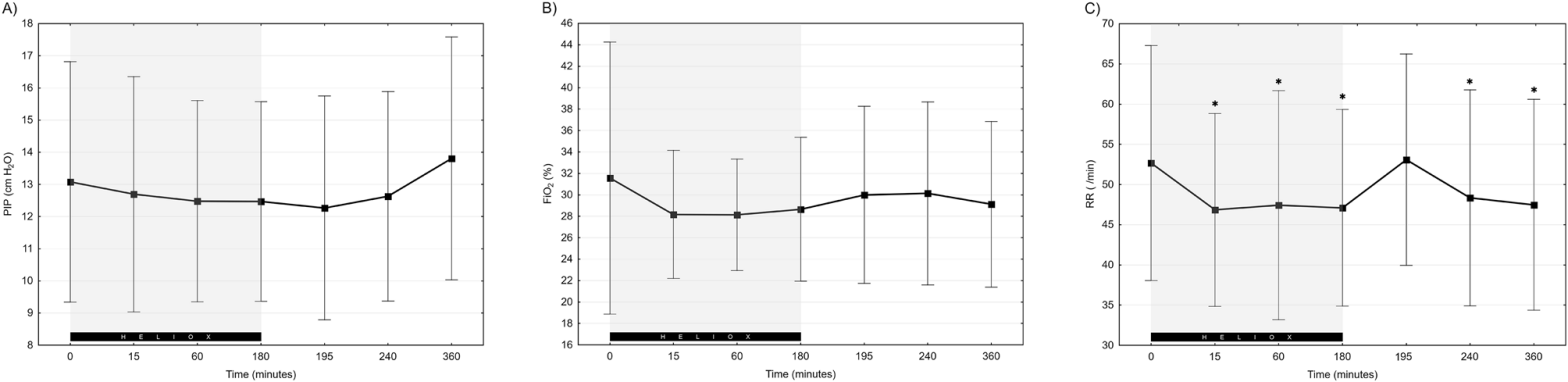
Ventilatory parameters during the study: PIP (**A**), FiO_2_ (**B**), RR (**C**). Grey background highlights heliox NIV-NAVA period. Data presented as mean ± SD; *indicates statistically significant changes versus baseline.

Capillary blood gas analysis did not reveal any significant differences between study periods – mean pH was 7.29 ± 0.04 vs 7.29 ± 0.04 vs 7.29 ± 0.04 and pCO_2_ 47.4 ± 11.6 vs 50.0 ± 8.8 vs 49.6 ± 9.5 [mm Hg] (*p* = 0.505). Ventilatory parameters and FiO_2_ did not differ between heliox and air-oxygen (Fig. 2A,B) (*p* = 0.476). Mean NIV leakage values were also similar (82.18 ± 17.17 vs 89.27 ± 4.92 [%], *p* = 0.572. NAVA level in the studied group was set between 1 and 4 cm H_2_O/μV (mean NAVA level = 1.55; SD = 1 [cm H_2_O/μV]). Heliox administration was associated with a rapid decrease in respiratory rate. After 15, 60 and 180 min of heliox ventilation RR was significantly lower than at beginning of the study (*p* = 0.017, *p* = 0.009 and *p* = 0.007, respectively). A temporary, increase in RR was observed immediately after disconnecting heliox (Fig. 2C). A significant difference was found between RR values recorded at 180 min of heliox and 15 min after return to air-oxygen ventilation (mean 47 vs 53; *p* = 0.019), however after 60 and 180 min of air-oxygen RR decreased and did not differ significantly as compared to values before heliox termination (mean 47 vs 48 and 47 respectively; *p* = 0.479 and 0.697).

**Figure 3:**
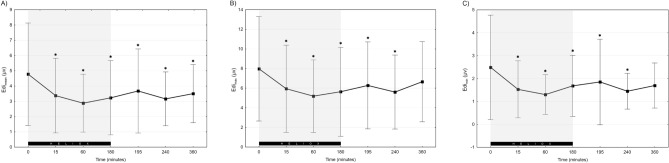
Electrical activity of the diaphragm during the study: Edi_mean_ (**A**), Edi_max_ (**B**), Edi_min_ (**C**). Grey background highlights heliox NIV-NAVA period. Data presented as mean ± SD; *indicates statistically significant changes versus baseline.

All measures of electrical activity of the diaphragm decreased after heliox administration. After 60’ of heliox particular Edi values were significantly lower than at baseline (Edi_min_ 1.3 vs 2.5 μV, *p* = 0.02; Edi_max_ 5.2 vs 8 μV, *p* = 0.002; Edi_mean_ 2.9 vs 4.8 μV, *p* < 0.001; Fig. 3A). Edi_min_ at the beginning of the study was significantly higher than at the further timepoints, except for the value at the end of the study when Edi_min_ started to rise (Fig. 3C). Edi_mean_ and Edi_min_ at the end of the protocol were significantly higher than in the 60^th^ minute after heliox NIV-NAVA (3.5 vs 2.9 [μV], *p* = 0.01; 1.7 vs 1.3 [μV] *p* = 0.03). However, the last recorded Edi_mean_ was significantly lower than values observed at baseline (4.8 vs 3.5 [μV], *p* = 0.024) (Fig. 3B).

In the subgroup analysis of primary heliox NIV-NAVA patients Edi_mean_ differed significantly from baseline at 60 and 180 min of heliox—3.87 vs 2.57 and 2.83 [μV], respectively (*p* = 0.008). Similar trends in Edi values were found in the post extubation subgroup, however observed differences were not significant.

Edi_max_ was significantly lower during heliox NIV-NAVA in comparison to the initial value. Discontinuation of heliox was associated with slow increase in Edi_max_, which at the 360th minute of the study was significantly higher than in the 60^th^ and 180^th^ minute (6.7 vs 5.2 and 5.6 [μV] respectively; *p* = 0.006, *p* = 0.02).

## Discussion

Ensuring the success of NIV in premature infants remains a major challenge to modern neonatology, as a considerable number of infants born prematurely fail early nasal continuous positive airway pressure (nCPAP)^17,18^. Once on mechanical ventilation, weaning may also present a difficulty, especially in the setting of developing bronchopulmonary dysplasia^19^. Heliox administration during non-invasive ventilation has been evaluated in a limited number of studies but it has been shown that it may be associated with certain clinical benefits. When combined with nCPAP, heliox was found to reduce the need for mechanical ventilation in preterm infants with respiratory distress syndrome in comparison with standard nCPAP^20^. Delivered with non-synchronized nasal intermittent positive pressure ventilation (NIPPV), heliox was effective in reducing the length of ventilation and increasing carbon dioxide elimination^21^. To our knowledge this is the first study assessing effects of heliox during NIV-NAVA in premature infants.

Few studies have assessed the effectiveness of heliox therapy on respiratory effort. Wolfson et al. reported that infants with bronchopulmonary dysplasia receiving heliox via face mask responded with a reduction in work of breathing (WOB) as measured by simultaneous pneumotachography and esophageal pressure^22^. A decrease in WOB was also revealed during heliox augmented mechanical ventilation^23^ and heliox delivery via nasal cannulae^24^. The present study confirms these observations using a new methodology, considering Edi as a surrogate for the WOB measurement. A study in an animal model found that increased diaphragm load, expressed by high voltage Edi_max_, is associated with damage to its sarcomeres^25^. In a porcine model of acute lung injury, high flow nasal cannula (HFNC) with heliox demonstrated a protective effect towards the diaphragm^26^. The significant and rapid decrease of Edi voltage after introduction of heliox suggests that the change of breathing mixture to heliox has direct influences on respiratory function. Absolute Edi values may be difficult to compare between different infants due to high variability, hence trends of this parameter seem most useful for assessment of changes in respiratory effort.

As potential rapid changes in partial pressure of CO_2_ due to improved gas flow and diffusion might influence cerebral blood flow StO_2_ monitoring during heliox with NIV-NAVA was performed. In this study no significant changes in StO_2_ were observed during heliox ventilation. However, patients included in the study were relatively stable and their baseline StO_2_ was within the normal range. Future studies should determine whether heliox might help to improve low StO_2_ in patients with severe respiratory failure. Although there are studies on heliox MV only single publications report on non-invasive respiratory support and the influence of the therapy on oxygenation in infants^27,28^. In the presented study the increase in SpO_2_ values after 1 h of heliox was statistically significant but very subtle—most likely of limited clinical importance. Withdrawal of heliox was associated with a significant decrease in SpO_2_, which confirms earlier observations reported by our group^3–5^.

In previous studies discontinuation of heliox after 1 h of mechanical ventilation was associated with significant deterioration of oxygenation and respiratory function suggesting that the duration of therapy might have been too short^3–5^. In the study by Dani et al., a decrease in MAP and pCO_2_ was found after 24 h of heliox^29^. Colnaghi et al. reported a reduction of non-invasive ventilation failure risk after 12-h of NIV with mixture of helium and oxygen^17^. We found the effect of heliox on Edi was observed after 15 min and the effect on SpO_2_ after one hour. After heliox NIV-NAVA was discontinued there was a trend towards return to pre-heliox Edi values. However, they did not reach the pre-intervention value, suggesting an improvement of respiratory function. The question remains whether prolonging the treatment with heliox beyond 3 h would further improve the clinical effects of the therapy. The minimum duration of successful treatment with neonatal heliox remains unknown and will likely depend on the underlying disease and the individual condition of each patient. The challenge in future studies will be to determine the threshold parameters for ending heliox therapy without the risk of deterioration. Edi decrement could serve as a potential predictor of the success of heliox therapy.

Application of NIV is associated with leak which can be influenced by the type of the interface used and its fitting. Based on theoretical assumptions regarding physical properties of heliox (low density and high diffusion coefficient) its use during NIV might be associated with increased leak as compared to air-oxgen. However, no significant difficulties regarding leak were observed in this study when heliox was delivered with NIV-NAVA using nasal masks. Effects of heliox were also described when it was used with high flow therapy during which high leak is routinely expected^23^. High leak during NIV makes flow-triggered synchronization challenging, however while Edi is used for triggering this is not a problem with NIV-NAVA.

Limitations of this pilot study include a small number of participants with relatively modest respiratory requirements and two types of NIV application: primary and post-extubation. Study protocol was not blinded, leading to a potential bias. Moreover, only selected physiologic parameters were analyzed over limited period of time, hence it is not possible to speculate about the long-term improvements in respiratory function or the impact of therapy on pulmonary outcomes. As suggested before there may be a synergistic effect of heliox and NIV-NAVA and it is difficult to assess their contribution to the observed effects. However, as NAVA level was kept constant during the study, decreasing Edi values after heliox administration and their increase observed following heliox discontinuation strongly suggest the influence of the gas mixture on the respiratory function.

Presented results of this pilot study warrant further research in a larger group of patients to better understand the potential synergistic effect of heliox and NIV-NAVA and the role of this combined therapy in enhancing the efficacy of non-invasive respiratory support in the preterm infants.

## Methods

An unblinded crossover pilot study was conducted in the NICU of the Department of Neonatology, Poznan University of Medical Sciences in Poznan, Poland. Infants eligible for inclusion were born ≤ 32 weeks gestational age (GA), had respiratory distress and required NIV as primary ventilation with fraction of inspired oxygen (FiO_2_) between 0.25 and 0.4 or secondary, after at least one failed extubation attempt. The latter were assessed by the treating physician as ready for another extubation according to specific criteria (spontaneous respiratory drive, mean airway pressure (MAP) < 8 cm H_2_O, capillary pH > 7.22 and pCO_2_ < 60 mmHg, post caffeine citrate administration); they received heliox NIV-NAVA within 1 h from extubation. Patients with major congenital defects, respiratory failure requiring mechanical ventilation or rescue surfactant administration were excluded from the trial. The study protocol was approved by the ethics committee of Poznan University of Medical Sciences. For all eligible infants written informed consent was obtained from their parents. The study has been performed in accordance with the Declaration of Helsinki and registered at ClinicalTrials.gov (NCT04404816, 28/05/2020).

Premature infants were first switched from non-synchronized NIPPV to NIV-NAVA using Maquet Servo-I ventilator (Getinge, Solna, Sweden). In the initial phase of the study NAVA level was optimized aiming at achieving peak inspiratory pressure (PIP) similar to values during preceding NIPPV, with positive end-expiratory pressure (PEEP) set at 5 cm H_2_O. When this was achieved recordings of 30 min were acquired at baseline while infants were ventilated with air-oxygen NIV-NAVA. Afterwards air was switched to heliox—mixture of 79% helium and 21% oxygen (Linde Group, Poland). After 3 h heliox was discontinued and the gas supply was changed back to the standard air-oxygen mixture. Ventilatory parameters, including NAVA level, were kept constant throughout the study. FiO_2_ was titrated to keep the SpO_2_ in the range of 90–95%. Monitoring of cerebral oxygenation (StO_2_), pulse oximeter oxygen saturation (SpO_2_) and heart rate (HR) were performed throughout the study using NONIN SenSmart Model X-100 (Nonin Medical Inc., Plymouth, USA). Selected ventilation parameters (PIP, PEEP, MAP, RR, NIV leakage), as well as Edi measures were recorded using Servo-tracker software (Getinge, Solna, Sweden). Blood gas analysis was performed in 3 study periods—at baseline, 3 h after heliox administration and 3 h after the return to standard gas mixture (Cobas b 221 blood gas system, Roche, Switzerland).

Analyses were performed using Statistica 12 (TIBCO Software, Palo Alto, California, USA) and PQStat v.1.6.8 software (PQStat Software, Poznan, Poland); *p* < 0.05 was considered significant. The normal distribution of variables was checked using the Shapiro–Wilk test. In order to examine changes in time, in the case of compliance with the normal distribution and equal variances, the variance analysis test was calculated for related samples. In the absence of compliance with a normal distribution the Friedman test was used. In the case of statistically significant differences in order to assess between which timepoints they occurred post hoc, Conover-Iman’s test was performed.

The datasets generated during and/or analyzed during the current study are available from the corresponding author on request.
